# Schistosomal Lipids Activate Human Eosinophils via Toll-Like Receptor 2 and PGD_2_ Receptors: 15-LO Role in Cytokine Secretion

**DOI:** 10.3389/fimmu.2018.03161

**Published:** 2019-01-25

**Authors:** Kelly G. Magalhães, Tatiana Luna-Gomes, Fabio Mesquita-Santos, Rafael Corrêa, Leonardo Santos Assunção, Georgia Correa Atella, Peter F. Weller, Christianne Bandeira-Melo, Patricia T. Bozza

**Affiliations:** ^1^Laboratório de Imunofarmacologia, Instituto Oswaldo Cruz, FIOCRUZ, Rio de Janeiro, Brazil; ^2^Laboratório Imunologia e Inflamação, Universidade de Brasília (UnB), Brasília, Brazil; ^3^Laboratório de Inflamação, Instituto de Biofísica Carlos Chagas Filho, Universidade Federal do Rio de Janeiro, Rio de Janeiro, Brazil; ^4^Departamento de Ciências da Natureza, Instituto de Aplicação Fernando Rodrigues da Silveira, Universidade do Estado do Rio de Janeiro, Rio de Janeiro, Brazil; ^5^Laboratório de Pesquisas em Análise Clínicas, Unidade de Farmácia, Centro Universitário da Zona Oeste, Rio de Janeiro, Brazil; ^6^Laboratório de Bioquímica de Lipídeos e Lipoproteínas, Universidade Federal do Rio de Janeiro, Rio de Janeiro, Brazil; ^7^Allergy and Inflammation, Harvard Medical School, Boston, MA, United States

**Keywords:** eosinophil, PGD_2_, lipids, schistosoma, TLR, LPC, TGFβ, eoxin

## Abstract

Parasite-derived lipids may play important roles in host-pathogen interactions and immune evasion mechanisms. Remarkable accumulation of eosinophils is a characteristic feature of inflammation associated with parasitic disease, especially caused by helminthes. Infiltrating eosinophils are implicated in the pathogenesis of helminth infection by virtue of their capacity to release an array of tissue-damaging and immunoregulatory mediators. However, the mechanisms involved in the activation of human eosinophils by parasite-derived molecules are not clear. Here we investigated the effects and mechanisms of schistosomal lipids-induced activation of human eosinophils. Our results showed that stimulation of human eosinophils *in vitro* with total lipid extracts from adult worms of *S. mansoni* induced direct activation of human eosinophils, eliciting lipid droplet biogenesis, synthesis of leukotriene (LT) C_4_ and eoxin (EX) C_4_ (14,15 LTC_4_) and secretion of eosinophil pre-formed TGFβ. We demonstrated that main eosinophil activating components within *S. mansoni* lipid extract are schistosomal-derived lysophosphatidylcholine (LPC) and prostaglandin (PG)D_2_. Moreover, TLR2 is up-regulated in human eosinophils upon stimulation with schistosomal lipids and pre-treatment with anti-TLR2 inhibited both schistosomal lipids- and LPC-, but not PGD_2_-, induced lipid droplet biogenesis and EXC_4_ synthesis within eosinophils, indicating that TLR2 mediates LPC-driven human eosinophil activation. By employing PGD_2_ receptor antagonists, we demonstrated that DP1 receptors are also involved in various parameters of human eosinophil activation induced by schistosomal lipids, but not by schistosomal LPC. In addition, schistosomal lipids and their active components PGD_2_ and LPC, triggered 15-LO dependent production of EXC_4_ and secretion of TGFβ. Taken together, our results showed that schistosomal lipids contain at least two components—LPC and PGD_2_—that are capable of direct activation of human eosinophils acting on distinct eosinophil-expressed receptors, noticeably TLR2 as well as DP1, trigger human eosinophil activation characterized by production/secretion of pro-inflammatory and immunoregulatory mediators.

## Introduction

Schistosomiasis is a chronic parasitic infection caused by five species of trematode helminths of the genus Schistosoma that infects more than 200 million people in developing countries (1). The major pathologic manifestations of the chronic *Schistosoma mansoni* disease are the eosinophil-enriched granulomatous response usually accompanied by severe hepatic and periportal fibrosis, portal hypertension, and portosystemic shunting of venous blood (2). *S. mansoni* are complex multicellular parasites that evolved some unique processes which are vital for their long-term survival within the mammalian host. This worm is capable of secreting molecules that subvert or suppress host immune responses and its cover tegument acts as an immune refractory barrier (1, 2). Schistosomal tegumental outer-surface structure appears to be critically involved in complex host–parasite interactions. Besides *S. mansoni* protein composition, schistosomal lipids have gained increased attention due to their important immunomodulatory properties (3–6). The most predominant phospholipid in *S. mansoni* cercariae, schistosomula and adults worms is phosphatidylcholine (7). *S. mansoni* lipids are required by the parasite not only to maintain its surface integrity and structural requirements but also for egg production, cell-cell signaling, and modulation of immune system (3, 8). Accordingly, employing a murine model of *S. mansoni* infection we have recently demonstrate that TLR2-dependent pathways activated *in vivo* by schistosomal-derived lipids play an important immunomodulatory role contributing to the pathogenesis and lethality in the chronic phase of infection (3, 4). Of note, we have shown that schistosomal-derived lipids, mostly lysophosphatidylcholine (LPC), were able to induce macrophage activation and polarization toward a M2 phenotype, and *in vivo* eosinophilic response (3, 4).

Eosinophils play an important role in modulating the host immune response to helminth infections (9), and eosinophilia has been largely recognized as a characteristic host response during schistosomiasis (10). Accumulating evidence has established eosinophils as multifunctional leukocytes with varied effector and immunomodulatory functions not only in allergic or helminthic disease but also in the initiation and amplification of numerous inflammatory and infectious responses and as modulators of innate and adaptive immunity (11). Although the roles of eosinophils as a defense mechanism against *S. mansoni* infection have been challenged and remain controversial (12–14), eosinophils may play modulatory roles in maintaining the Th2 response to infection via cytokine secretion, and may contribute to the cytokine-mediated pathogenesis (15, 16). Here we hypothesized that parasite-derived lipids may play roles in host-pathogen interactions by activating eosinophils to release immunomodulatory and pro-fibrotic mediators.

## Materials and Methods

### Purification and Analysis of *S. mansoni* Lipids

The total lipid extracts were isolated from adult worms of *S. mansoni* and lipids were extracted as describe (3). Briefly, lipids from *S. mansoni* worms were extracted for 2 h with a chloroform-methanol-water solution (2:1:0.8, v/v). After centrifugation, the supernatant was collected and the pellet subjected to a second lipid extraction for 1 h. Schistosomal total lipids (Schisto-TL) were used to stimulate eosinophils (see below) or were subjected to two dimensional thin-layer chromatography (TLC) for phospholipid fractionation and analysis and lysophospholipid (LPC) extraction. Schistosomal LPCs (Schisto-LPC) were removed from TLC using the method previously described (3). The composition and purity of schistosomal derived-LPC fractions were analyzed by ES-MS/MS and by GC/MS. As previously demonstrated (3), Schisto-LPC contained principally the fatty acids palmitic acid (m/z 518.3 = 16:0 LPC [M + Na]+; m/z 496.3 = 16:0- LPC [M + H]+) or stearic acid (m/z 546.3 = 18:0-LPC [M + Na]+; m/z 524.3 = 18:0-LPC [M + H]+). The analysis of the mass spectra both on positive and negative modes (not shown) confirmed the purity of the LPC fraction. The LPC species on schistosomal-derived samples were further confirmed by GC/MS. Together LPC C16:0 and LPC C18:0 comprises over 94% of the LPC species identified in 4 independent purifications of schisto-derived LPC.

### Isolation of Human Blood Eosinophils

Peripheral blood was obtained with informed consent from healthy donors. Briefly, after dextran sedimentation and Ficoll gradient steps, eosinophils were isolated from contaminating neutrophils by negative immunomagnetic selection using the EasySep™ system (StemCell Technologies Inc.)(cell purity ~99%; cell viability ~95%) (17). The protocol was approved by ethical review boards of the Beth Israel Deaconess Medical Center Committee on Clinical Investigation and Federal University of Rio de Janeiro (Rio de Janeiro, Brazil).

### *In vitro* Stimulation of Human Blood Eosinophils

Human eosinophils (2 × 10^6^ cells/mL) were incubated in Ca^2+^/Mg^2+^ HBSS (HBSS^+/+^; pH 7.4) for 1 h (37°C) with schistosomal total lipids extract (Schisto-TL; 1 μg/mL); purified schistosomal LPC (Schisto-LPC; 0.01 or 0.1 μg/mL), arachidonic acid (AA; 10 μM), PGD_2_ (5 or 25 nM), a combination of LPC (0.01 μg/mL) and PGD_2_ (5 nM), EXC_4_ (0.03–3 μM) or LTC_4_ (0.03–3 μM). AA, PGD_2_, EXC_4_, and LTC_4_ were from Cayman Chemicals. For mechanistic studies, eosinophils were pretreated for 30 min with a TLR2 neutralizing antibody (1 μg/mL; Invitrogen), an inhibitor of 15-LO-1 enzymatic activity (15-lipoxygenase inhibitor 1, 200 nM; Cayman Chemical), or selective antagonists of DP1 (BWA868c, 200 nM; Cayman Chemicals) and (Cay 10471, 200 nM; Cayman Chemicals) DP2 receptors prior to stimulation. Each experiment was repeated at least three times with eosinophils purified from different donors.

### Lipid Droplet Staining and Enumeration

Lipid droplets were stained and enumerated as previously described (18). For lipid droplet enumeration within eosinophil cytoplasm, cytospin cells were fixed in 3.7% formaldehyde (diluted in HBSS^−/−^), rinsed in 0.1 M cacodylate buffer (pH 7.4), stained with 1.5% OsO_4_ for 30 min, rinsed in distilled H_2_O, immersed in 1.0% thiocarbohydrazide for 5 min, rinsed in 0.1 M cacodylate buffer, restained with 1.5% OsO_4_ for 3 min, rinsed in distilled water, and mounted. Lipid droplets were enumerated by light (osmium staining) microscopy. Fifty consecutively scanned eosinophils were evaluated in a blinded fashion by more than one individual, and the results were expressed as the number of lipid droplets *per* eosinophil.

Alternatively, analysis of lipid droplets was performed with Nile Red (Sigma-Aldrich) for better visualization of the cytoplasmic distribution of eosinophil lipid droplets. Briefly, while still moist, eosinophils on cytospin slides were fixed in 3.7% formaldehyde in HBSS^−/−^ pH 7.4, rinsed with PBS buffer and then incubated with Nile Red for 30 min, rinsed in dH_2_O, incubated with DAPI (4′,6-Diamidino-2-phenylindole dihydrochloride) (Sigma-Aldrich) per 5 min, rinsed in dH_2_O, and then dried and mounted.

### Expression of Perilipin 2 (PLIN2)/Adipose Differentiation-Related Protein (ADRP) and 15-LO

Analysis of PLIN2/ADRP and 15-LO expression within human eosinophils were done by western blotting. In brief, eosinophil lysates were prepared in reducing and denaturing conditions and subjected to SDS-PAGE. Samples were submitted to electrophoresis in 5–15% acrylamide gradient SDS-PAGE gels. After transfer onto nitrocellulose membranes, non-specific binding sites were blocked with 5% non-fat milk in Tris buffered saline-Tween (TBST; 50 mM Tris-HCl, pH 7.4, 150 mM NaCl, 0.05% Tween 20). Membranes were probed with guinea pig polyclonal ADRP antibody (AP 002; Fitzgerald, MA); anti-15-LO-1 (Cayman Chemical), and anti-β-actin mAb (BD Transduction Laboratories) in TBST with 1% non-fat dry milk. Proteins of interest were then identified by incubating the membrane with HRP-conjugated secondary antibodies in TBST, followed by detection of antigen-antibody complexes by Supersignal Chemiluminescence (Pierce). The detection was done by exposing membranes to autoradiography film.

### TLR2 Expression

Analysis of TLR2 expression was performed by flow cytometry analysis. Human eosinophils were washed with HBSS^−/−^ and then incubated for 30 min with FITC-conjugated anti-TLR2 mAb (clone TL 2.1) or IgG2a k isotype control-FITC from eBioscience. After washings, cells were analyzed by flow cytometry in a FACS Calibur (BD Biosciences) flow cytometer.

### EicosaCell for Intracellular EXC_4_ Immunodetection

EicosaCell technique (19) was used to immunodetect EXC_4_ at its intracellular synthesis sites. *In vitro*-stimulated human eosinophils were mixed with an equal volume of water-soluble 1-ethyl-3-(3-dimethylamino-propyl) carbodiimide (EDAC; 0.2% in HBSS containing 1% BSA for 10 min) (Sigma), used to cross-link eicosanoid carboxyl groups to amines in adjacent proteins. Eosinophils, washed, cytospun onto glass slides and subjected to a blocking step (1%BSA for 30 min), were incubated with rabbit anti-EXC_4_ Abs (Cayman Chemicals) overnight and secondary DyLight488 green fluorochrome anti-rabbit IgG (Jackson ImmunoResearch Laboratories) for 1 h. As specificity controls for the immunolocalization of EXC_4_, rabbit IgG (Sigma) was routinely included as a non-immune control for the primary anti-EXC_4_ (with no detectable staining; data not shown). Mounting medium containing DAPI was applied to each slide before coverslip attachment to allow visualization of blue-stained eosinophil nuclei. Images were obtained using an Olympus BX51 fluorescence microscope at 100x magnification and photographs were taken with the Olympus 72 digital camera (Olympus Optical Co., Tokyo, Japan) in conjunction with CellF Imaging Software for Life Science Microscopy (Olympus Life Science Europa GMBH, Hamburg, Germany).

### Eicosanoid Quantification

LTC_4_ or EXC_4_ (14, 15 LTC_4_) found in eosinophil supernatants and PGD_2_ found in schistosomal lipid extracts were measured by specific commercial EIA kits, according to the manufacturer's instructions (Cayman).

### Analysis of TGFβ Secretion

Cell-free supernatants from *in vitro* stimulated eosinophils were collected and stored at −20°C until the day of analysis. Human TGFβ were measured by commercial ELISA kits, according to the manufacturer's instructions (R&D Systems).

In addition to the study of TGFβ release, the intracellular TGFβ levels were assessed by flow cytometry. Briefly, eosinophils were stimulated for 1 h with Schisto-TL (1μg/mL), Schisto-LPC (0.1μg/mL) or vehicle. After that, cells were fixed in 4% paraformaldehyde fixative (PFA), washed with PBS and incubated for 15 min with permeabilization buffer (saponin 0.05% in PBS containing 2% BSA). Cells were then washed and incubated for 30 min with FITC-labeled goat anti-TGFβ Ab from eBioscience (or FITC-labeled isotype) diluted in permeabilization buffer. After washings, cells were analyzed by flow cytometry in a FACSCalibur flow cytometer (BD Biosciences).

### Statistical Analysis

Data are expressed as mean ± SEM of at least three independent experiments. Multiple comparisons among groups were performed by one-way ANOVA followed by Student-Newman-Keuls test, with the level of significance set at *p* < 0.05.

## Results

### Schistosomal Lipids Directly Activate Lipid Droplet Biogenesis in Human Eosinophils in a TLR2 Dependent Manner

**Figure 1:**
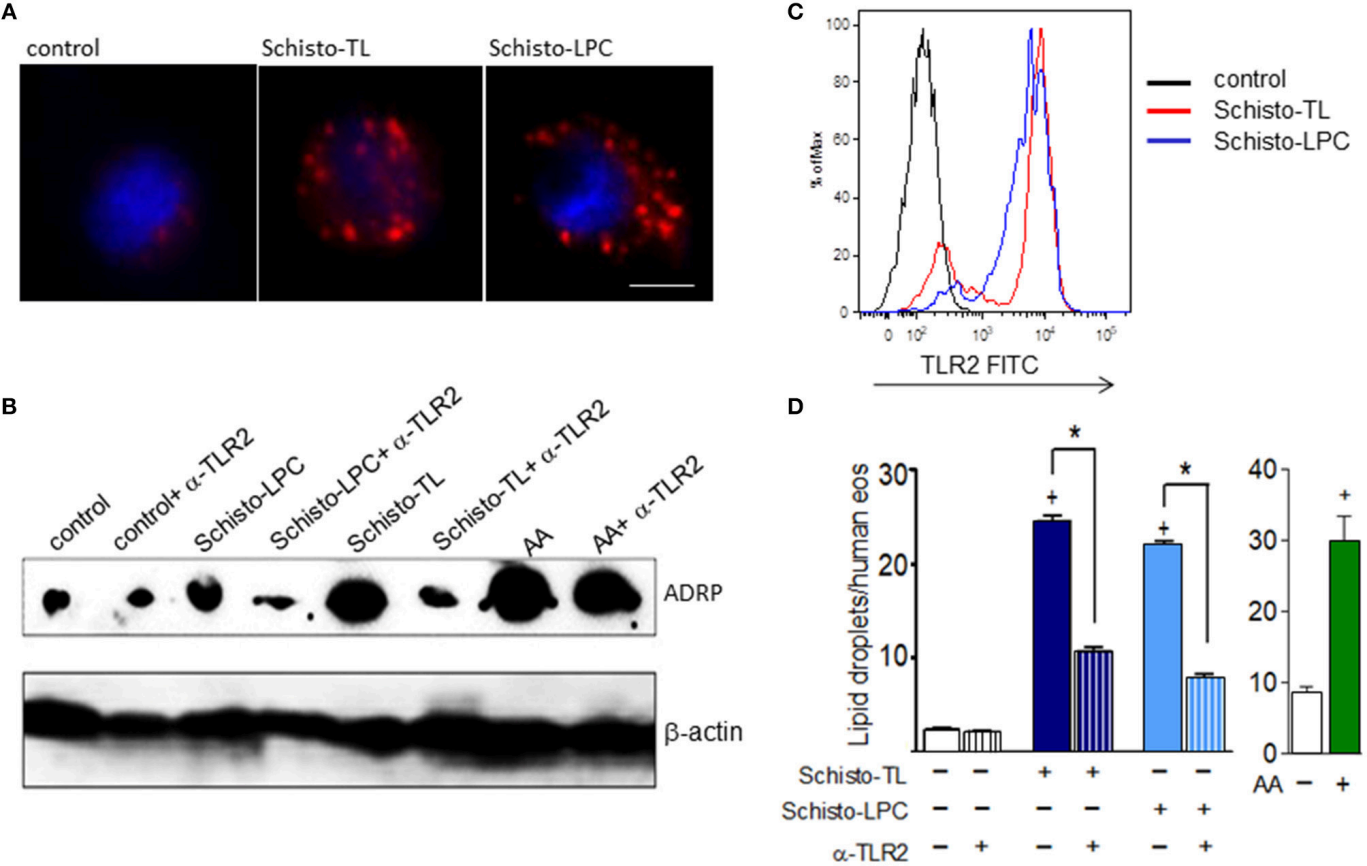
Schistosomal total lipids and LPC purified fractions triggers assembly of new lipid droplets within human eosinophils via TLR2 activation. Human eosinophils were pretreated with neutralizing anti-TLR2 antibody (as indicated) 30 min before stimulation with Schisto-TL (1 μg/mL), Schisto-LPC (0.1 μg/mL), or AA (10 μM) for 1 h. **(A)** Shows the cytoplasmic distribution of lipid droplets stained by Nile Red within human eosinophils. **(B)** Shows the intracellular expression of the lipid droplet protein marker ADRP evaluated by Western blot of eosinophil lysates. In **(C)**, Increases in surface expression of TLR2 were detected by flow cytometry. In **(D)**, lipid droplets were enumerated in 50 consecutive osmium-stained cells. Values are expressed as the mean ± SEM of at least three distinct donors. +*p* < 0.05 compared with non-stimulated eosinophils. *p < 0.05 compared with lipid-stimulated eosinophils.

To verify if schistosomal lipids could directly trigger eosinophil activation, we isolated human eosinophils and stimulated them with schistosomal lipid extract (Schisto-TL) or purified schistosomal-derived lysophosphatidylcholine fraction (Schisto-LPC). Increased numbers of lipid droplets in eosinophils have been considered as markers of cell activation and have been associated with increased capacity of eosinophil eicosanoid synthesis (20, 21). As shown in Figure 1, schistosomal lipids as well as purified schistosomal-derived LPC triggered significant increases in lipid droplet numbers and increased expression of the lipid droplet structural protein and marker PLIN2/ADRP after 1 h (Figures 1A,B,D) when compared to non-stimulated eosinophils. Since schistosomal lipids were described as a TLR2 signaling pathway activators in murine infections (3), we checked if human eosinophils were able to express TLR2. The results demonstrated that human eosinophils constitutively express cell surface TLR2, which was upregulated after stimulation with Schisto-TL or Schisto-LPC as assessed by FACS analysis (Figure 1C). Similarly, TLR2 mRNA levels assessed by RT-PCR were also upregulated by schistosomal lipids (Supplementary Figure 1). To confirm that TLR2 was playing a role in the lipid droplet biogenesis triggered by Schisto-TL and Schisto-LPC in eosinophils, we blocked TLR2 with a neutralizing antibody, and analyzed lipid droplet biogenesis as well as the levels of ADRP. Our results indicated that lipid droplet biogenesis and ADRP expression triggered by Schito-TL and Schisto-LPC occurred in a TLR2-dependent manner (Figures 1B,D).

### Eosinophil Lipid Droplet Biogenesis Elicited by *S. mansoni*-Derived Lipids Is Also Mediated by DP1 Activation

**Figure 2:**
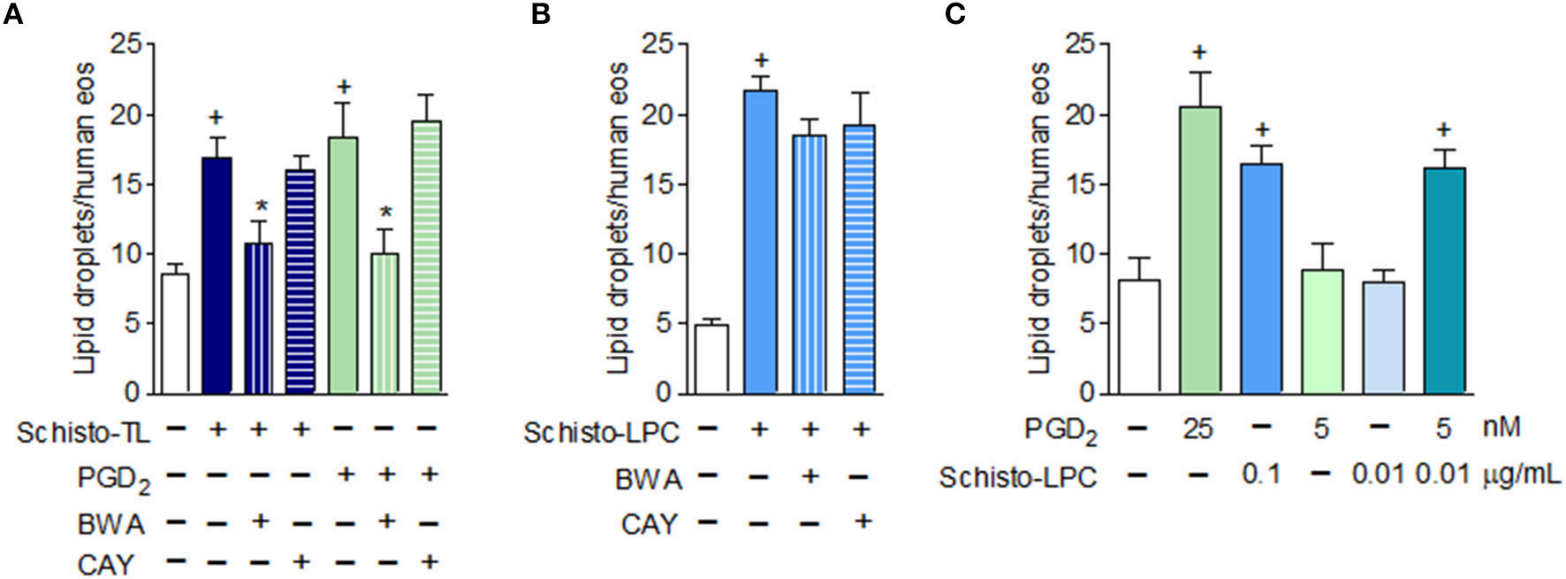
Schistosomal lipid, other than LPC, triggers lipid droplet biogenesis within human eosinophils via activation of PGD_2_ receptor DP1. In **(A)**, human eosinophils were pretreated with DP1 (BWA868c; 200 nM) or DP2 (Cay10471; 200 nM) antagonists 30 min before stimulation with Schisto-TL (1 μg/mL) or PGD_2_ (25 nM) for 1 h. In **(B)**, human eosinophils were pretreated with DP1 (BWA868c; 200 nM) or DP2 (Cay10471; 200 nM) antagonists 30 min before stimulation with Schisto-LPC (0.1 μg/mL) for 1 h. **(C)** Shows synergism effect of human eosinophils stimulated with PGD_2_ (5 nM) and Schisto-LPC (0.01 μg/mL) for 1 h. Lipid droplet counts were evaluated in osmium-stained cells. Values are expressed as the mean ± SEM of at least three distinct donors. +*p* < 0.05 compared with non-stimulated cells. *p < 0.05 compared with lipid-stimulated eosinophils.

Since it has been demonstrated that the helminth *S. mansoni* is capable of producing PGD_2_ (22), we wondered if lipid droplet biogenesis triggered by Schisto-TL and Schisto-LPC could be mediated by PGD_2_ receptors, DP1 and DP2, in addition to TLR2. To address this question, we treated human eosinophils with DP1 receptor antagonist BWA868c and DP2 receptor antagonist Cay10471 prior to stimulation with Schisto-TL and Schisto-LPC. Our results showed that schistosomal TL triggered DP1 dependent but DP2 independent lipid droplet biogenesis (Figure 2A). Of note, eosinophils stimulated with PGD_2_ display similar DP1 dependent- but DP2 independent lipid droplet biogenesis, as previously reported [Figure 2A and (23)]. In addition, treatment with an anti-TLR2 neutralizing antibody, while effective against Schisto-LPC (Figure 1D), failed to inhibit lipid droplet formation induced by PGD_2_ (from 17.67 ± 0.10 to 19.5 ± 0.2 lipid droplets/eosinophil in PGD_2_ compared to anti-TLR2 plus PGD_2_; non-significant).

We also verified if LPC could trigger lipid droplet biogenesis in eosinophils signaling through DP1 receptors. However, different to what was observed with Schisto-TL-stimulated eosinophils, Schisto-LPC triggered DP1-independent lipid droplet formation (Figure 2B). Additionally, we showed that PGD_2_ induced lipid droplet formation in a dose-dependent manner, and we observed a synergistic effect in lipid droplet formation when eosinophils were stimulated along with subliminal concentrations of both PGD_2_ and LPC (Figure 2C). Of note, the presence of PGD_2_ in the schistosomal lipid extract was confirmed by EIA (~150 pg/mL of PGD_2_/Schisto-TL), indicating that Schisto-TL effects on eosinophils may be due to synergistic interactions between both lipids PGD_2_ and Schsito-LPC present in the total lipid extract of *S. mansoni*.

### Schistosomal-Derived Lipids Induce Leukotriene C_4_ (LTC_4_) and EXC_4_ (14,15 LTC_4_) Generation by Human Eosinophils

**Figure 3:**
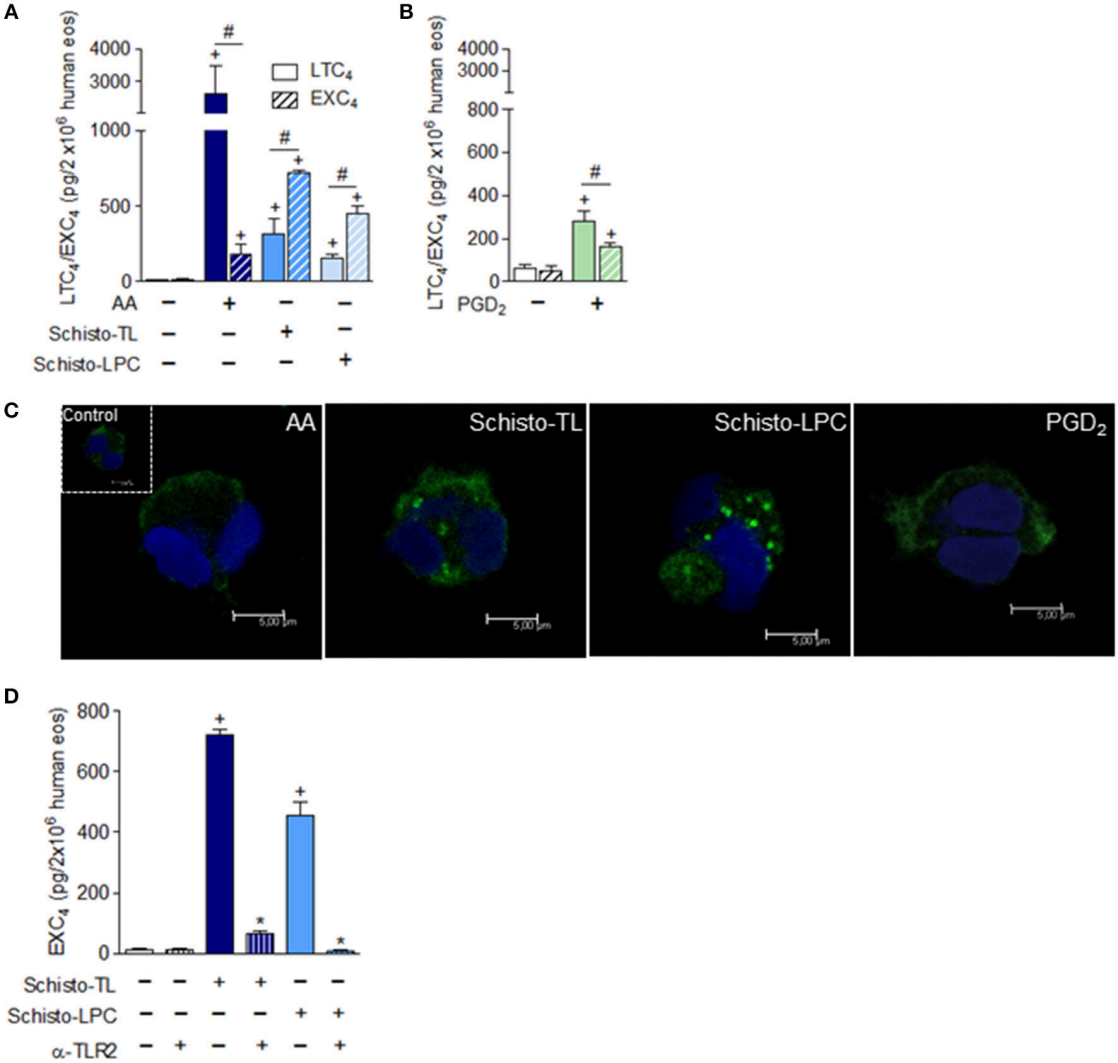
Differential synthesis of LTC_4_ vs. EXC_4_ triggered by schistosomal bioactive lipids within human eosinophils. In **(A)**, human eosinophils were stimulated with Schisto-TL (1 μg/mL), Schisto-LPC (0.1 μg/mL), or AA (10 μM) for 1 h. In **(B)**, human eosinophils were stimulated with PGD_2_ (25 nM) for 1 h. **(C)** Shows confocal images overlays of intracellular EicosaCell immuno-detection of newly formed EXC_4_ (green) and DAPI stained nuclei (blue) within AA, Schisto-TL-, Schisto-LPC-or PGD_2_-stimulated human eosinophils. In **(D)**, human eosinophils were pretreated with neutralizing anti-TLR2 antibody 30 min before stimulation with Schisto-TL (1 μg/mL) or Schisto-LPC (0.1 μg/mL) for 1 h. **(A,B,D)** Show LTC_4_ and/or EXC_4_ production in cell-free supernatants quantified by specific EIA kits. Values are expressed as the mean ± SEM of at least three distinct donors. +*p* < 0.05 compared with non-stimulated cells. *p < 0.05 compared with lipid-stimulated eosinophils. #*p* < 0.05 as indicated.

Considering that lipid droplets are major intracellular sites involved in eicosanoid synthesis during inflammatory conditions (21) and eosinophils are an abundant source of 5-LO and 15-LO derived lipid mediators, we analyzed if Schisto-TL and Schisto-LPC could activate generation of 5-LO-derived LTC_4_ and 15-LO-derived EXC_4_ by human eosinophils. Our results showed that both Schisto-TL and Schisto-LPC triggers LTC_4_ and EXC_4_ synthesis by human eosinophils (Figure 3A). Different from AA- or PGD_2_-stimulated eosinophils for which LTC_4_ is the major leukotriene formed within eosinophils, the relative amounts of EXC_4_ induced by Schisto-TL and Schisto-LPC were higher than LTC_4_ amounts (Figures 3A,B). The increased EXC_4_ levels induced by Schisto-TL and Schisto-LPC in comparison with AA stimulation could be also visualized intracellularly in human eosinophils as assessed by Eicosacell assay (Figure 3C). Detailed analysis revealed that most newly synthesized EXC_4_ (green labeling) within Schisto-TL-, Schisto-LPC, and PGD_2_-stimulated eosinophils was in a punctate cytoplasmic pattern proximate to, but separate from, the nucleus and fully consistent in size and form with eosinophil lipid droplets (Figure 3C).

In order to understand which receptor could be playing a role in EXC_4_ generation by Schisto-TL and Schisto-LPC, human eosinophils were pre-treated with TLR2 blocking antibody prior to stimulation with Schisto-TL and Schisto-LPC. The results demonstrated that EXC_4_ secretion induced by Schisto-TL or by Schisto-LPC occurred in a TLR2- dependent manner (Figure 3D).

### Schistosomal-Derived Lipids Trigger 15-LO Dependent EXC_4_ Generation and Preformed TGFβ Release by Human Eosinophils

**Figure 4:**
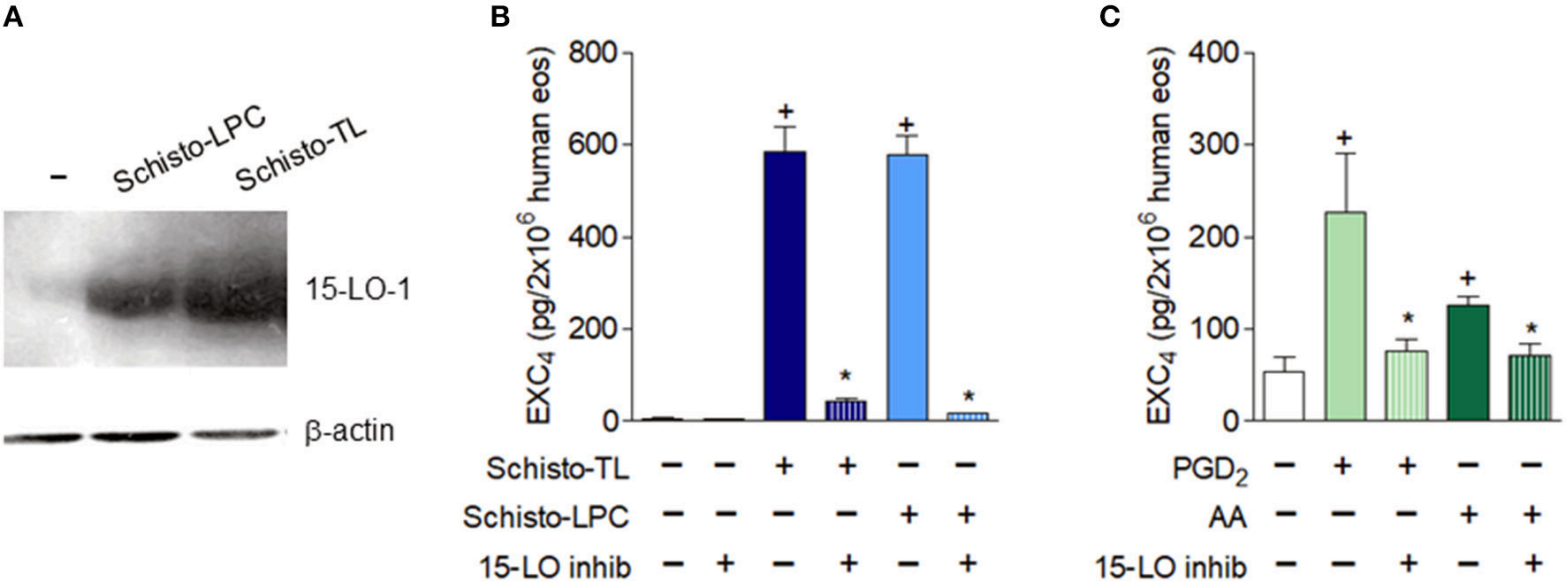
Schistosomal lipids promote up-regulated expression and activation of the EXC_4_-synthesizing enzyme 15-LO within human eosinophils. **(A)** Shows intracellular expression of the 15-LO protein evaluated by Western blot of cell lysates of human eosinophils stimulated with Schisto-TL (1 μg/mL) or Schisto-LPC (0.1 μg/mL) for 1 h. In **(B,C)**, human eosinophils were pretreated with inhibitor of 15-LO enzymatic activity 30 min before stimulation with Schisto-TL (1 μg/mL), Schisto-LPC (0.1 μg/mL), PGD_2_ (25 nM) or AA (10 μM) for 1 h. EXC_4_ contents in cell-free supernatants were evaluated by specific EIA kit. Values are expressed as the mean ± SEM of at least three distinct donors. +*p* < 0.05 compared with non-stimulated cells. *p < 0.05 compared with lipid-stimulated eosinophils.

Human eosinophils constitutively express 15-LO, which can be further upregulated upon activation (24–26). We first analyzed if Schisto-TL and Schisto-LPC could modulate eosinophil 15-LO expression. Our data showed that both Schisto-TL and Schisto-LPC induced significant increases of 15-LO-1 expression in human eosinophils (Figure 4A). Pre-treatment with a selective 15-LO inhibitor demonstrated that EXC_4_ synthesis triggered by Schisto-TL and its two components studied here, Schisto-LPC and PGD_2_, is dependent on 15-LO enzymatic activity within eosinophils, inasmuch as the 15-LO inhibitor blocked EXC_4_ synthesis within Schisto-TL, Schisto-LPC, PGD_2_, or AA-stimulated eosinophils (Figures 4B,C).

**Figure 5:**
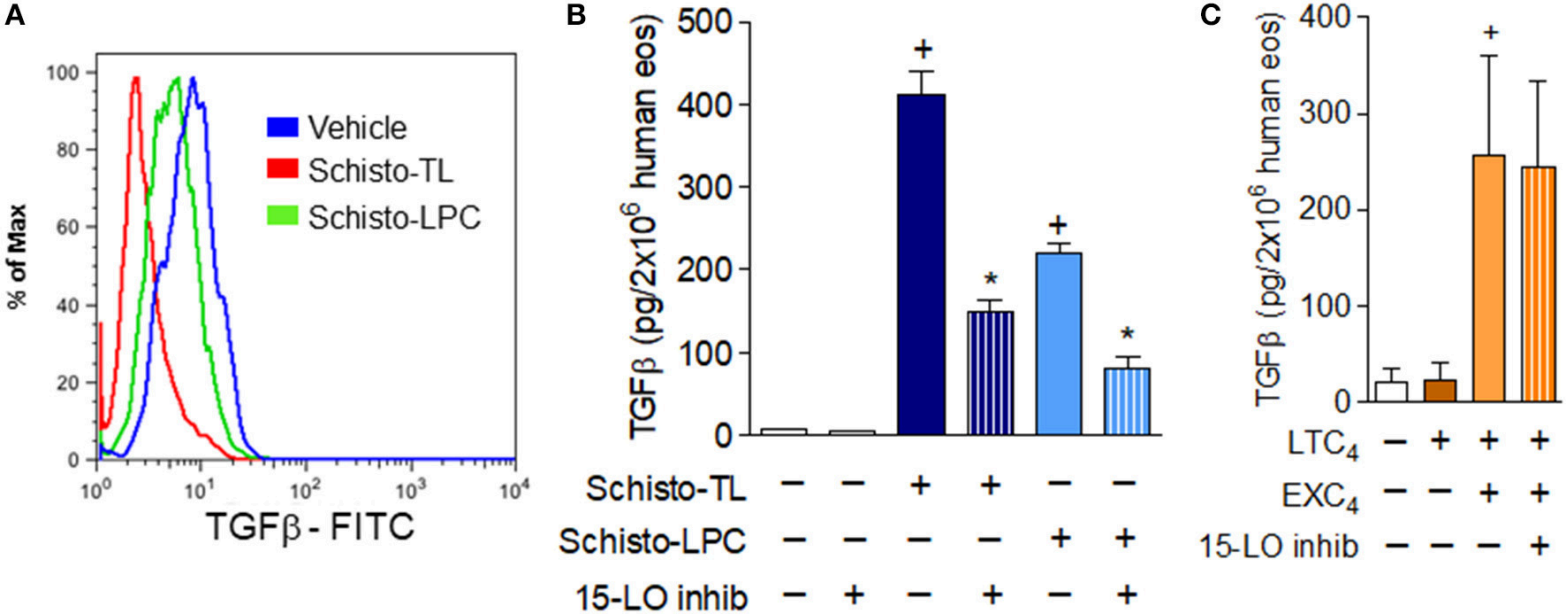
15-LO-driven EXC_4_ synthesis mediates schistosomal lipids-induced rapid TGFβ secretion from human eosinophils. **(A)** Shows decreases in intracellular expression of TGFβ detected by flow cytometry within human eosinophils stimulated with Schisto-TL (1 μg/mL) or Schisto-LPC (0.1 μg/mL) for 1 h. In **(B)**, human eosinophils were pretreated with an inhibitor of 15-LO enzymatic activity 30 min before stimulation with Schisto-TL (1 μg/mL) or Schisto-LPC (0.1 μg/mL) for 1 h. In **(C)**, human eosinophils were pretreated with inhibitor of 15-LO enzymatic activity 30 min before stimulation with EXC_4_ (0.3 μM) or LTC_4_ (0.3 μM) for 1 h. TGFβ content in cell-free supernatants were evaluated by specific ELISA kit. Values are expressed as the mean ± SEM of at least three distinct donors. +*p* < 0.05 compared with non-stimulated cells. *p < 0.05 compared with lipid-stimulated eosinophils.

Eosinophils store pro-fibrogenic cytokines including TGFβ (27) in their intracellular granules. Among schistosomiasis-related eosinophil functions, secretion of such fibrogenic cytokines as TGF-β appear to have roles in this hepatic granulofibrotic disease (28, 29). Stimulation of human eosinophils with Schisto-TL and Schisto-LPC triggered rapid (within 1 h of stimulation) TGFβ secretion, as assessed by both decrease of intracellular preformed stores of TGFβ by flow cytometry (Figure 5A) and increase of released extracellular levels of TGFβ in human eosinophil supernatants (Figure 5B).

In order to study potential roles of eosinophil 15-LO-driven EXC_4_ synthesis on TGFβ release from human eosinophils, we analyzed whether 15-LO inhibitor could impair schistosomal lipids-induced secretion of preformed TGFβ from human eosinophils. As shown in Figure 5B, our data indicated that both Schisto-TL- or Schisto-LPC-triggered TGFβ secretion were dependent of 15-LO activity, since 15-LO inhibition reduced eosinophil extracellular levels of TGFβ. As shown in Table 1, while either EXC_4_ or LTC_4_ failed to induce lipid droplet biogenesis within eosinophils, stimulation of human eosinophils by EXC_4_, but not by LTC_4_, lead to increased TGFβ release by human eosinophils (Figure 5C). Of note, EXC_4_-triggered TGFβ secretion was not modified by 15-LO inhibition (Figure 5C), therefore supporting EXC_4_-mediated autocrine activity of 15-LO-driven TGFβ release by human eosinophil stimulated with schistosomal lipids.

**Table 1:** *In vitro* stimulation with EXC_4_ is not able to trigger lipid droplet biogenesis within human eosinophils^a^.

**Condition**	**[μM]**	**Lipid droplets/eosinophil**
–		12.6 ± 3.1
EXC_4_	0.03	14.9 ± 2.2
	0.3	8.0 ± 3.0
	3	11.5 ± 1.7
LTC_4_	0.03	16.2 ± 6.5
	0.3	15.8 ± 4.4
	3	14.8 ± 6.5
AA	10	28.0 ± 2.1^+^

a *Human eosinophils were incubated for 1 h with different concentrations of EXC_4_, LTC_4_ or AA (as indicated). Lipid droplets were enumerated in 50 consecutive osmium-stained cells. Values are expressed as the mean ± SEM of at least three distinct donors. +p < 0.05 compared with non-stimulated eosinophils*.

### Discussion

Eosinophils are known to display defensive and pro-inflammatory activities in a variety of helminth infections. However, rather than effector cells of helminthiasis, eosinophils have recently emerged as immunomodulatory cells capable of acting to maintain homeostasis, resolve inflammation, promote Th2 immune responses, and repair damaged tissues, by inducing, for instance, secretion of pro-fibrotic mediators like TGFβ. While mediators known to activate the effector functions of eosinophils in helminth infection-associated eosinophilia are mostly host-derived molecules, direct stimulation of eosinophils with helminth-derived molecules is poorly or not studied and may trigger such immunomodulatory eosinophils. Stimulatory molecules that trigger fine-tuned eosinophil activation characterized by secretion of eosinophil-derived molecules with pro-resolving/repairing, pro-fibrotic impacts remain largely uncharacterized, here; we showed that schistosomal derived lipids can directly stimulate human eosinophils to secrete pro-fibrotic TGFβ.

**Figure 6:**
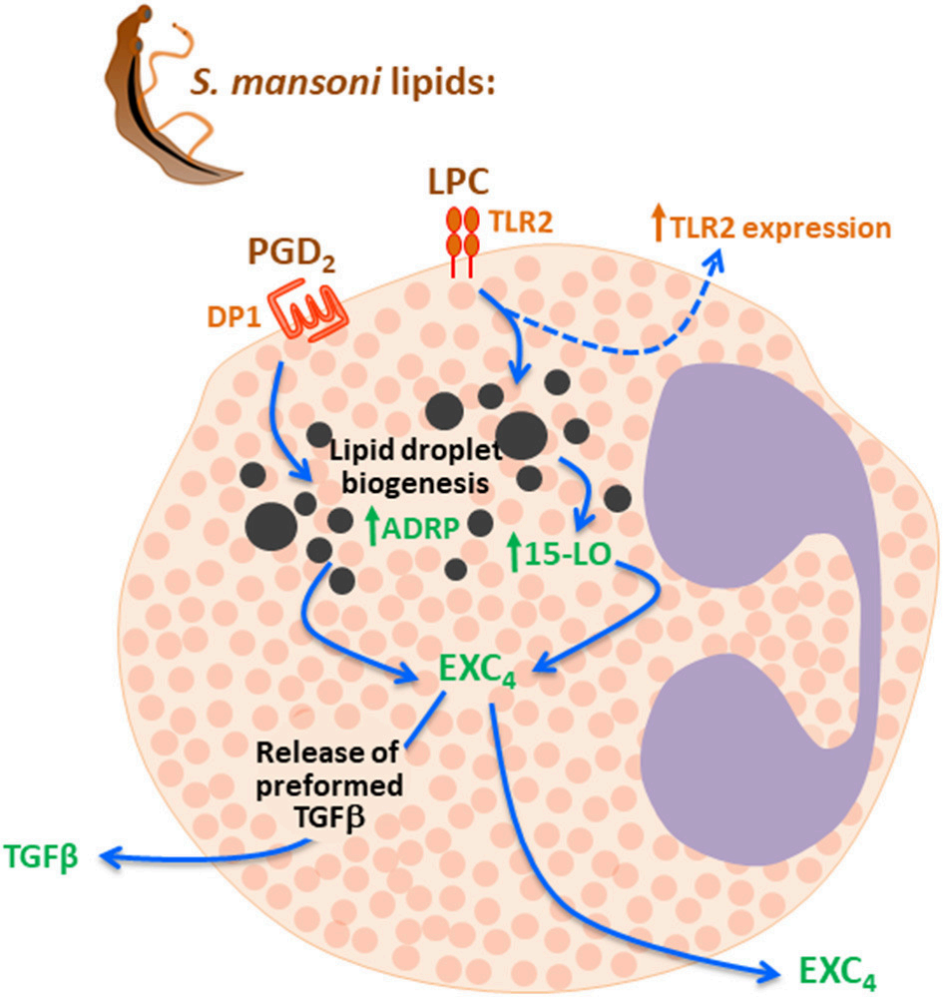
Schistosomal lipids LPC and PGD_2_ trigger 15-LO-driven EXC_4_ synthesis via TLR2 and DP1 activation which culminates with TGF-β secretion. Schistosomal total lipids stimulation of eosinophils activates at least two receptors on eosinophils membranes, including TLR2 and DP1, due agonistic effect of LPC and PGD_2_ present in the lipid extract. Then, both LPC and PGD2 are able to evoke lipid droplet biogenesis, activation of 15-LO enzyme within these lipidic organelles and lipid-droplet-compartmentalized EXC_4_ synthesis. Although levels of EXC_4_ are secreted by eosinophils under schistosomal lipids stimulation, newly synthesized EXC_4_ may act intracellularly to trigger the rapid TGF-β secretion observed.

Here, we demonstrated that schistosomal lipids exhibit distinctive regulatory functions in activating arachidonic acid metabolism and cytokine release from human eosinophils. Both schistosomal-derived LPC and PGD_2_ acting on specific membrane receptors, TLR2 and DP1 respectively, activated a 15-LO-driven intracellular pathway promoting lipid droplet-compartmentalized EXC_4_ synthesis and rapid secretion of eosinophil preformed TGFβ (Figure 6). Full eosinophil secretory capability upon stimulation with schistosomal lipids (including LPC, PGD_2_ and any other bioactive schistosomal lipid not studied here) are far from fully characterized. Nevertheless, our study is pioneer in demonstrating that schistosomal lipids are indeed capable of directly activating secretion of immunomodulatory/pro-fibrotic molecules from human eosinophils.

Our current findings demonstrate that schistosomal LPC and PGD_2_, are recognized, respectively, by TLR2 and DP1 receptors on human eosinophils, to trigger secretion of at least three active molecules: EXC_4_, LTC_4_, and TGFβ from eosinophils. While the last two eosinophil-derived molecules are well recognized mediators of eosinophil responses in several eosinophilic conditions as helminth infections, EXC_4_ is a more recently described eicosanoid produced in high quantities by eosinophils through the 15-LO pathway associated with asthma (30, 31). For EXC_4_ there is no receptor identified to date and virtually no cellular activity has been described. Exception is the *in vitro* demonstration that, similar to receptor-mediated LTC_4_-induced effect, EXC_4_ is able to directly increase cell permeability of human endothelial cells (30). Specifically regarding human eosinophil activation and again similar to LTC_4_ (32), EXC_4_ does not have the ability to trigger lipid droplet biogenesis and therefore may be unable to assemble the intracellular compartments of eicosanoid synthesis within eosinophils. On the other hand, EXC_4_ was capable of inducing TGFβ secretion from human eosinophils (or mouse eosinophils; data not shown), showing that EXC_4_ stimulation is not species-specific and triggers a specific pattern of eosinophil activation compatible with a receptor-initiated event. While other eosinophil stimulatory functions triggered by exogenous or eosinophil-derived 15-LO-driven EXC_4_ are still pending characterization, our data shows that EXC_4_ synthesis and TGFβ secretion are mediated by eosinophil 15-LO activity. In agreement, 15-LO has been show to participate in the regulation of cytokine expression and release in other cell types (33), in addition to involvement in the biosynthesis of pro-resolving lipids, such as lipoxins (31), resolvins (34), maresins (35), and protectins (36). Based in a history of autocrine (or even intracrine) activities of eosinophil-derived eicosanoids mediating eosinophil functions (37), as well as, EXC_4_ ability to induce TGFβ secretion from eosinophils seen here, it would be reasonable to postulate that 15-LO-driven synthesized EXC_4_ under schistosomal lipids-stimulation mediates subsequent TGFβ secretion by eosinophils. Further assays are needed to confirm such hypothesis, inasmuch as other 15-LO metabolites could participate in TGFβ release. Unfortunately, studies of putative autocrine activities of eosinophil-derived EXC_4_ are still hampered by the lack of receptor identification and, therefore antagonist availability. On the other hand, the partial involvement of 15-LO on TGFβ secretion from eosinophils (since inhibition of 15-LO activity did not block completely TGFβ secretion) indicates that besides 15-LO-driven eosinophil-derived molecules, 15-LO-independent eosinophil-derived mediators secreted by eosinophils upon schistosomal-derived lipids activation may also be regulating TGFβ secretion. Among 15-LO-independent eosinophil-derived molecules secreted upon schistosomal lipids-stimulation, we have discarded 5-LO metabolite LTC_4_ as an autocrine/paracrine mediator of schistosomal lipids-induced TGFβ secretion, since in contrast to EXC_4_, LTC_4_ failed to trigger TGFβ secretion from eosinophils. Although LTC_4_ does not control TGFβ secretion by human eosinophils, cross-talks between leukotrienes and TGFβ have been appreciated in a variety of cell sources, including stellate hepatic cells, macrophages and eosinophils (38–42). In addition, concerning *S. mansoni* infection where IL-4-mediated Th2 immune response play major roles in pathology (2), LTC_4_ can function as an eosinophil-derived signal that promotes IL-4 secretion from eosinophils (43, 44). Accordingly, studies employing 5-LO deficient animals have demonstrated an immune-regulatory role for 5-LO in schistosomal infection by the demonstration of decreased levels of IL-4 and IL-13, and decreased granuloma size in 5-LO deficient animals, while exhibiting increased levels of TGFβ and fibrosis (45, 46).

We have previously shown that schistosomal-derived lipid LPC induces *in vivo* eosinophil recruitment and activation in a murine model of *S. mansoni* infection via TLR2-initiated signaling pathways (3). Moreover, TLR2 expression on human eosinophils have been already shown, however its activation is reported to trigger eosinophil effector functions, such as eosinophil degranulation of cytotoxic ECP protein and superoxide production (47), rather than immunomodulatory ones. More consonant with our data showing LPC/TLR2-driven secretion of immunomodulatory molecules from human eosinophils, as recently reported, schistosomal LPC can induce macrophage activation and polarization toward the anti-inflammatory M2 phenotype with TGFβ secretion (4).

Schistosomiasis is a major intravascular infection, and adult worm live in direct contact with the mesenteric or portal vein endothelium. Adult worms continuously expose, secrete and excrete numerous substances that may directly or indirectly activate eosinophils in the liver and eosinopoiesis in the bone marrow and/or extramedullary. Here we demonstrate that adult worm lipid extracts contain at least two molecules, LPC and PGD_2_, capable to directly activate human through TLR-2 and DP1 pathways, respectively. Adult worms also produce large numbers of intravascular eggs, and indeed schistosomal trapped eggs in the host tissue have important pathological roles in eosinophil-granulomatous inflammatory response, raising the intriguing question as whether schistosomal eggs have similar eosinophil activation capacity to directly activating secretion of immunomodulatory/pro-fibrotic molecules from human eosinophils, through TLR-2 and DP1 pathways. Of note, recent lipidome characterization of *S. mansoni* has demonstrated that egg-derived lipids from *S. mansoni* exhibit similar content of LPC and PGD_2_ as the adult worm (48). Moreover, egg-derived lipids activate murine macrophages through TLR2-dependent pathways as observed for adult worms lipid extracts (3). As such, similar direct eosinophil activation is expect to occur with egg-derived lipids, but further experiments will be necessary to fully address this question.

Regarding DP1 activation by PGD_2_ on eosinophils, in addition to prior studies by our group that have shown its ability to promote lipid droplet-driven 5-LO-mediated LTC_4_ synthesis, the current study is the first to show 15-LO activation by PGD_2_. Of note, besides eosinophils and virtually all other immune cells, DP1 is widely expressed on tissues, including the vasculature, the central nervous system, the retina, and the lungs (49–52). Therefore, schistosomal PGD_2_ has the potentiality to affect a variety of physiological functions in the host tissues. Even though PGD_2_ has been initially perceived as a pro-inflammatory mediator, the exact function of DP1 activation has not been fully elucidated yet, but the understanding of PGD_2_/DP1 functions are now evolving to a more immunomodulatory/pro-resolving type similar to eosinophils themselves (53). For instance, by activating DP1 receptors, PGD_2_ is known to inhibit the functions of platelet, neutrophils, basophils, and dendritic cells (54). In agreement, based on a study employing *in vivo* model of *S. mansoni* infection in DP1 deficient mice, authors have postulated that DP1 activation by *S. mansoni*-derived PGD_2_ represent a schistosomal strategy to evade host immune defenses via altered balance of Th1/Th2 immune responses (55). In human eosinophils, activation of DP1 is not down-regulatory *per se*, however it appears to evoke primarily secretion of potential down-modulators. It is noteworthy that LPC and PGD_2_ molecules are lipids that can also be produced by host cells during *S. mansoni* infection. For instance, we have shown that *S. mansoni* infection-derived stellate hepatic cells are able to synthesize LTC_4_ and PGD_2_, which control in an autocrine fashion TGFβ release (40, 56). Similarly, commercial LPC has been shown to modulate secretion of TGFβ by different cell types (4, 57).

In conclusion, our findings provide original evidence that schistosomal lipids contain at least two components that are capable of direct activation of human eosinophils, LPC and PGD_2_ that through receptor-mediated response trigger eosinophil activation characterized by rapid release of pro-inflammatory and pro-fibrotic mediators. The identification of bioactive schistosomal lipids and mapping the functional impact of these lipids on eosinophils are germane to better understanding the schistosomal pathology, as well as, may lead to new insights into improved treatment for both schistosomiasis and other eosinophilic immunological diseases.

## Author Contributions

KM, PW, CB-M, and PB conceived and designed the study. KM designed and performed the experiments, analyzed, and interpreted data. PB, CB-M, and KM wrote and revised the manuscript. TL-G, FM-S, LA, RC, and GA participated in the data acquisition, analysis, and interpretation. All authors had critically revised and approved the final version of the manuscript.

### Conflict of Interest Statement

The authors declare that the research was conducted in the absence of any commercial or financial relationships that could be construed as a potential conflict of interest.

## Supplementary Material

Supplementary Figure 1 Schistosomal lipids promote up-regulation of TLR2 mRNA expression within human eosinophils. The image shows TLR2 mRNA expression in human eosinophil as assessed by RT-PCR. Human eosinophils were stimulated with Pam3Cys (300 ng/ml), Schisto-TL (1 μg/mL) or Schisto-LPC (0.1 μg/mL) for 1 h. Primer sequences for human hTLR2 were used from TLR2 detection kit (Invivogen) and human GAPDH were used as housekeeping control.

## References

[1] van der Kleij D Yazdanbakhsh M. Control of inflammatory diseases by pathogens: lipids and the immune system. Eur J Immunol. (2003) 33:2953–63. 10.1002/eji.200324340 14579263

[2] Pearce EJ MacDonald AS. The immunobiology of schistosomiasis. Nat Rev Immunol. (2002) 2:499–511. 10.1038/nri843 12094224

[3] Magalhães KG Almeida PE Atella GC Maya-monteiro CM Castro-faria-neto HC Pelajo-machado M Schistosomal-derived lysophosphatidylcholine are involved in eosinophil activation and recruitment through toll-like receptor – 2 – dependent mechanisms. J Infect Dis. (2010) 900:1369–79. 10.1086/656477 20863227

[4] Assunção LS Magalhães KG Carneiro AB Molinaro R Almeida PE Atella GC. Schistosomal-derived lysophosphatidylcholine triggers M2 polarization of macrophages through PPARγ dependent mechanisms. Biochim Biophys Acta Mol Cell Biol Lipids (2017) 1862:246–54. 10.1016/j.bbalip.2016.11.006 27871882

[5] Van Hellemond JJ Retra K Brouwers JFHM van Balkom BWM Yazdanbakhsh M Shoemaker CB. Functions of the tegument of schistosomes: clues from the proteome and lipidome. Int J Parasitol. (2006) 36:691–9. 10.1016/j.ijpara.2006.01.007 16545817

[6] Van der Kleij D Latz E Brouwers JFHM Kruize YCM Schmitz M Kurt-Jones EA. A novel host-parasite lipid cross-talk. Schistosomal lyso-phosphatidylserine activates toll-like receptor 2 and affects immune polarization. J Biol Chem. (2002) 277:48122–9. 10.1074/jbc.M206941200 12359728

[7] Furlong ST Caulfield JP. Schistosoma mansoni: sterol and phospholipid composition of cercariae, schistosomula, and adults. Exp Parasitol. (1988) 65:222–31. 10.1016/0014-4894(88)90126-9 3350102

[8] Van Der Kleij D Tielens AGM Yazdanbakhsh M. Recognition of schistosome glycolipids by immunoglobulin E: possible role in immunity. Infect Immun. (1999) 67:5946–50. 10531252 10.1128/iai.67.11.5946-5950.1999 PMC96978

[9] Rothenberg ME Hogan SP. THE EOSINOPHIL. Annu Rev Immunol. (2006) 24:147–74. 10.1146/annurev.immunol.24.021605.090720 16551246

[10] Klion AD Nutman TB. The role of eosinophils in host defense against helminth parasites. J Allergy Clin Immunol. (2004) 113:30–7. 10.1016/j.jaci.2003.10.050 14713904

[11] Weller PF Spencer LA. Functions of tissue-resident eosinophils. Nat Rev Immunol (2017) 17:746–60. 10.1038/nri.2017.95 28891557 PMC5783317

[12] Strandmark J Rausch S Hartmann S Eosinophils in homeostasis and their contrasting roles during inflammation and helminth infections. Crit Rev Immunol. (2016) 17:746–60. 10.1615/CritRevImmunol.2016018726 28008805

[13] Sher A Coffman RL Hieny S Cheever AW. Ablation of eosinophil and IgE responses with anti-IL-5 or anti-IL-4 antibodies fails to affect immunity against Schistosoma mansoni in the mouse. J Immunol. (1990) 145:3911–6. 10.1016/j.avb.2015.09.014 2123226

[14] Swartz JM Dyer KD Cheever AW Ramalingam T Pesnicak L Domachowske JB. Schistosoma mansoni infection in eosinophil lineage-ablated mice. Blood (2006) 108:2420–7. 10.1182/blood-2006-04-015933 16772607 PMC1895572

[15] Sabin EA Kopf MA Pearce EJ. Schistosoma mansoni egg-induced early IL-4 production is dependent upon IL-5 and eosinophils. J Exp Med. (1996) 184:1871–8. 10.1084/jem.184.5.1871 8920874 PMC2192874

[16] Rumbley CA Sugaya H Zekavat SA El Refaei M Perrin PJ Phillips SM. Activated eosinophils are the major source of Th2-associated cytokines in the schistosome granuloma. J Immunol. (1999) 162:1003–9. 9916726

[17] Luna-Gomes T Magalhaes KG Mesquita-Santos FP Bakker-Abreu I Samico RF Molinaro R. Eosinophils as a novel cell source of prostaglandin D2: autocrine role in allergic inflammation. J Immunol. (2011) 187:6518–26. 10.4049/jimmunol.1101806 22102725 PMC3237921

[18] Melo RCN D'Ávila H Bozza PT Weller PF. Imaging lipid bodies within leukocytes with different light microscopy techniques. In: Chiarini-Garcia H, Melo RCN, editors. Methods in Molecular Biology. Totowa, NJ: Humana Press. p. 149–61. 10.1007/978-1-60761-950-5_9 PMC3659330 21153791

[19] Bandeira-Melo C Paiva LA Amorim NRT Weller PF Bozza PT. Eicosacell: an imaging-based assay to identify spatiotemporal eicosanoid synthesis. In: Kalyuzhny A. Editor. Signal Transduction Immunohistochemistry. Methods in Molecular Biology, Vol. 1554. New York, NY: Humana Press (2017). p. 127–41. 10.1007/978-1-4939-6759-9_6 PMC5774667 28185186

[20] Bozza PT Magalhães KG Weller PF. Leukocyte lipid bodies – biogenesis and functions in inflammation. Biochim Biophys Acta Mol Cell Biol Lipids (2009) 1791:540–51. 10.1016/j.bbalip.2009.01.005 19416659 PMC2693476

[21] Bozza PT Bakker-Abreu I Navarro-Xavier RA Bandeira-Melo C. Lipid body function in eicosanoid synthesis: an update. Prostaglandins Leukot Essent Fat Acids (2011) 85:205–13. 10.1016/j.plefa.2011.04.020 21565480

[22] Angeli V Faveeuw C Roye O Fontaine J Teissier E Capron A. Role of the parasite-derived prostaglandin D2 in the inhibition of epidermal Langerhans cell migration during schistosomiasis infection. J Exp Med. (2001) 193:1135–47. 10.1084/jem.193.10.1135 11369785 PMC2193325

[23] Mesquita-Santos FP Bakker-Abreu I Luna-Gomes T Bozza PT Diaz BL Bandeira-Melo C. Co-operative signalling through DP(1) and DP(2) prostanoid receptors is required to enhance leukotriene C(4) synthesis induced by prostaglandin D(2) in eosinophils. Br J Pharmacol. (2011) 162:1674–85. 10.1111/j.1476-5381.2010.01086.x 20973774 PMC3081113

[24] Sigal E Sloane DL Conrad DJ. Human 15-lipoxygenase: induction by interleukin-4 and insights into positional specificity. J Lipid Mediat. (1993) 6:75–88. 8358018

[25] Bozza PT Yu W Cassara J Weller PF. Pathways for eosinophil lipid body induction: differing signal transduction in cells from normal and hypereosinophilic subjects. J Leukoc Biol. (1998) 64:563–9. 10.1002/jlb.64.4.563 9766638

[26] Archambault AS Turcotte C Martin C Provost V Larose MC Laprise C Comparison of eight 15-lipoxygenase (LO) inhibitors on the biosynthesis of 15-LO metabolites by human neutrophils and eosinophils. PLoS ONE (2018) 2018:e0202424 10.1371/journal.pone.0202424 PMC6097673 30118527

[27] Wong DT Elovic A Matossian K Nagura N McBride J Chou MY. Eosinophils from patients with blood eosinophilia express transforming growth factor beta 1. Blood (1991) 78:2702–7. 1726708

[28] Kamdem SD Moyou-Somo R Brombacher F Nono JK Cytokine regulation of schistosome-induced granuloma and fibrosis. Kidney Int. (1997) 51:1370–75. 10.1038/ki.1997.187 9150446

[29] Kamdem SD Moyou-Somo R Brombacher F Nono JK. Host regulators of Liver fibrosis during Human Schistosomiasis. Front Immunol. (2018) 9:2781. 10.3389/fimmu.2018.02781 30546364 PMC6279936

[30] Feltenmark S Gautam N Brunnstrom A Griffiths W Backman L Edenius C. Eoxins are proinflammatory arachidonic acid metabolites produced via the 15-lipoxygenase-1 pathway in human eosinophils and mast cells. Proc Natl Acad Sci. (2008) 105:680–5. 10.1073/pnas.0710127105 18184802 PMC2206596

[31] Sachs-Olsen C Sanak M Lang AM Gielicz A Mowinckel P Lødrup Carlsen KC. Eoxins: a new inflammatory pathway in childhood asthma. J Allergy Clin Immunol. (2010) 126:859–67.e9. 10.1016/j.jaci.2010.07.015 20920774

[32] Bozza PT Yu W Penrose JF Morgan ES Dvorak a M Weller PF. Eosinophil lipid bodies: specific, inducible intracellular sites for enhanced eicosanoid formation. J Exp Med. (1997) 186:909–20. 10.1084/jem.186.6.909 9294145 PMC2199047

[33] Wen Y Gu J Chakrabarti SK Aylor K Marshall J Takahashi Y. The role of 12/15-lipoxygenase in the expression of interleukin-6 and tumor necrosis factor-α in macrophages. Endocrinology (2007) 148:1313–22. 10.1210/en.2006-0665 17170102

[34] Yoo S Lim JY Hwang SW. Resolvins: endogenously-generated potent painkilling substances and their therapeutic perspectives. Curr Neuropharmacol. (2013) 11:664–76. 10.2174/1570159X11311060009 24396341 PMC3849791

[35] Serhan CN Dalli J Colas RA Winkler JW Chiang N. Protectins and maresins: new pro-resolving families of mediators in acute inflammation and resolution bioactive metabolome. Biochim Biophys Acta Mol Cell Biol Lipids (2015) 1851:397–413. 10.1016/j.bbalip.2014.08.006 25139562 PMC4324013

[36] Serhan CN Petasis NA. Resolvins and protectins in inflammation resolution. Chem Rev. (2011) 111:5922–43. 10.1021/cr100396c 21766791 PMC3192290

[37] Amorim NRT Luna-Gomes T Gama-Almeida M Souza-Almeida G Canetti C Diaz BL. Leptin elicits LTC4 synthesis by eosinophils mediated by sequential two-step autocrine activation of CCR3 and PGD2 receptors. Front Immunol. (2018) 9:1–11. 10.3389/fimmu.2018.02139 30298073 PMC6160734

[38] Wong DT Elovic A Matossian K Nagura N McBride J Chou MY. Eosinophils from patients with blood eosinophilia express transforming growth factor beta 1. Blood (1991) 78:2702–7. 1726708

[39] Elovic AE Ohyama H Sauty A McBride J Tsuji T Nagai M. IL-4-dependent regulation of TGF-alpha and TGF-beta1 expression in human eosinophils. J Immunol. (1998) 160:6121–7. 9637529

[40] Paiva LA Maya-Monteiro CM Bandeira-Melo C Silva PMR El-Cheikh MC Teodoro AJ. Interplay of cysteinyl leukotrienes and TGF-β in the activation of hepatic stellate cells from Schistosoma mansoni granulomas. Biochim Biophys Acta Mol Cell Biol Lipids (2010) 1801:1341–8. 10.1016/j.bbalip.2010.08.014 20817008

[41] Esser J Gehrmann U D'Alexandri FL Hidalgo-Estévez AM Wheelock CE Scheynius A. Exosomes from human macrophages and dendritic cells contain enzymes for leukotriene biosynthesis and promote granulocyte migration. J Allergy Clin Immunol. (2010) 126:1032–40. 10.1016/j.jaci.2010.06.039 20728205

[42] Freire-de-Lima CG Yi QX Gardai SJ Bratton DL Schiemann WP Henson PM. Apoptotic cells, through transforming growth factor-β, coordinately induce anti-inflammatory and suppress pro-inflammatory eicosanoid and NO synthesis in murine macrophages. J Biol Chem. (2006) 281:38376–84. 10.1074/jbc.M605146200 17056601

[43] Bandeira Melo C Woods LJ Phoofolo M Weller PF. Intracrine cysteinyl leukotriene receptor-mediated signaling of eosinophil vesicular transport-mediated interleukin-4 secretion. J Exp Med. (2002). 10.1084/jem.20020516 12235216 PMC2194050

[44] Bandeira-Melo C Hall JC Penrose JF Weller PF Cysteinyl leukotrienes induce IL-4 release from cord blood-derived human eosinophils. J Allergy Clin Immunol. (2002)196:841–50. 10.1067/mai.2002.124269 12063527

[45] Secor WE Powell MR Morgan J Wynn TA Funk CD Mice deficient for 5-lipoxygenase, but not leukocyte-type 12- lipoxygenase, display altered immune responses during infection with Schistosoma mansoni. Prostaglandins Other Lipid Mediat. (1998) 56:291–30. 10.1016/S0090-6980(98)00059-8 9990674

[46] Toffoli da Silva G Espíndola MS Fontanari C Rosada RS Faccioli LH Ramos SG. 5-lipoxygenase pathway is essential for the control of granuloma extension induced by Schistosoma mansoni eggs in lung. Exp Parasitol. (2016) 167:124–9. 10.1016/j.exppara.2016.06.001 27262746

[47] Wong CK Cheung PFY Ip WK Lam CWK. Intracellular signaling mechanisms regulating toll-like receptor–mediated activation of eosinophils. Am J Respir Cell Mol Biol. (2007) 37:85–96. 10.1165/rcmb.2006-0457OC 17332440

[48] Giera M Kaisar MMM Derks RJE Steenvoorden E Kruize YCM Hokke CH. The Schistosoma mansoni lipidome: leads for immunomodulation. Anal Chim Acta (2018) 1037:107–18. 10.1016/j.aca.2017.11.058 30292284

[49] Wright DH Ford-Hutchinson AW Chadee K Metters KM. The human prostanoid DP receptor stimulates mucin secretion in LS174T cells. Br J Pharmacol. (2000) 131:1537–45. 10.1038/sj.bjp.0703688 11139429 PMC1572485

[50] Nantel F Fong C Lamontagne S Wright DH Giaid A Desrosiers M. Expression of prostaglandin D synthase and the prostaglandin D2 receptors DP and CRTH2 in human nasal mucosa. Prostaglandins Other Lipid Mediat. (2004) 73:87–101. 10.1016/j.prostaglandins.2003.12.002 15165034

[51] Mohri I Kadoyama K Kanekiyo T Sato Y Kagitani-Shimono K Saito Y. Hematopoietic prostaglandin D synthase and DP1 receptor are selectively upregulated in microglia and astrocytes within senile plaques from human patients and in a mouse model of alzheimer disease. J Neuropathol Exp Neurol. (2007) 66:469–80. 10.1097/01.jnen.0000240472.43038.27 17549007

[52] Gerashchenko D Beuckmann CT Kanaoka Y Eguchi N Gordon WC Urade Y. Dominant expression of rat prostanoid DP receptor mRNA in leptomeninges, inner segments of photoreceptor cells, iris epithelium, and ciliary processes. J Neurochem. (1998) 71:937–45. 9721719 10.1046/j.1471-4159.1998.71030937.x

[53] Kostenis E Ulven T. Emerging roles of DP and CRTH2 in allergic inflammation. Trends Mol Med. (2006) 12:148–58. 10.1016/j.molmed.2006.02.005 16545607

[54] Peinhaupt M Sturm EM Heinemann A. Prostaglandins and their receptors in eosinophil function and as therapeutic targets. Front Med. (2017) 4:1–12. 10.3389/fmed.2017.00104 28770200 PMC5515835

[55] Hervé M Angeli V Pinzar E Wintjens R Faveeuw C Narumiya S. Pivotal roles of the parasite PGD2synthase and of the host D prostanoid receptor 1 in schistosome immune evasion. Eur J Immunol. (2003) 33:2764–72. 10.1002/eji.200324143 14515260

[56] Paiva LA Coelho KA Luna-Gomes T El-Cheikh MC Borojevic R Perez SA Schistosome infection-derived Hepatic Stellate Cells are cellular source of prostaglandin D2: role in TGF-β-stimulated VEGF production. Prostaglandins Leukot Essent Fat Acids (2015) 95:57–62. 10.1016/j.plefa.2015.01.004 25687497

[57] Zhou SX Huo DM He XY Yu P Xiao YH Ou CL High glucose/lysophosphatidylcholine levels stimulate extracellular matrix deposition in diabetic nephropathy via platelet-activating factor receptor. Mol Med Rep. (2018) 17:2366–72. 10.3892/mmr.2017.8102 29207067 PMC5783481

