# Supplementary figures and images for: Schistosomal Lipids Activate Human Eosinophils via Toll-Like Receptor 2 and PGD2 Receptors: 15-LO Role in Cytokine Secretion
Source: Front Immunol. 2019 Jan 25;9:3161. doi: 10.3389/fimmu.2018.03161 (PMC6355688; doi:10.3389/fimmu.2018.03161)

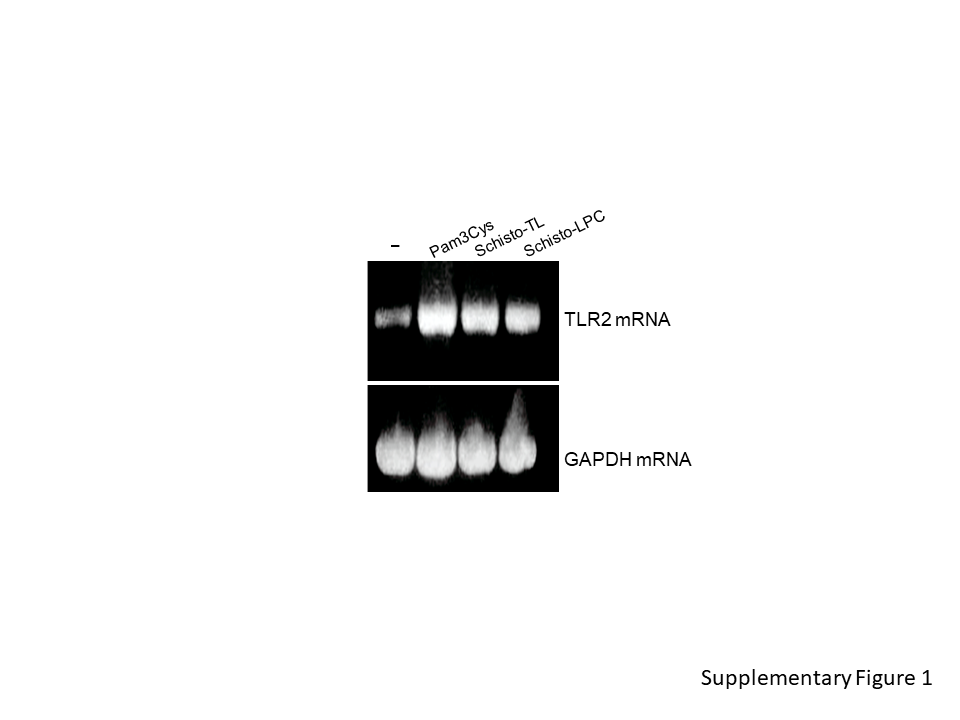

Supplement: Supplementary Figure 1 — Schistosomal lipids promote up-regulation of TLR2 mRNA expression within human eosinophils. The image shows TLR2 mRNA expression in human eosinophil as assessed by RT-PCR. Human eosinophils were stimulated with Pam3Cys (300 ng/ml), Schisto-TL (1 μg/mL) or Schisto-LPC (0.1 μg/mL) for 1 h. Primer sequences for human hTLR2 were used from TLR2 detection kit (Invivogen) and human GAPDH were used as housekeeping control. [file Image_1.TIF]
